# Knowledge and Attitude About Epilepsy Among School Teachers in Madinah, Saudi Arabia

**DOI:** 10.7759/cureus.44572

**Published:** 2023-09-02

**Authors:** Bayan Almarwani, Elaaf Alqelaiti, Ajyal Aljohani, Leen Abuanq, Rafa Alhujaili, Renas Aljohani

**Affiliations:** 1 Neurology, Taibah University, Madinah, SAU; 2 College of Medicine, Taibah University, Madinah, SAU

**Keywords:** epilepsy, knowledge, attitudes, neurological disorder, schoolteachers, saudi arabia

## Abstract

Background and objective: Epilepsy is one of the most common chronic neurological disorders affecting school-age children. School teachers play an essential role in the life and development of patients with epilepsy. Hence, adequate knowledge of and positive attitudes toward epilepsy among school teachers are critical. This study assessed the knowledge of and attitude toward epilepsy among primary, middle, and high school teachers in Madinah, Saudi Arabia.

Method: This cross-sectional study was conducted using a self-administered electronic questionnaire to assess the knowledge and attitude about epilepsy among 394 teachers from local schools.

Results: Most of the participants were female (64.7%). About 32.2% of the participants had experience teaching children with epilepsy. The mean score for knowledge about epilepsy was 8.2 out of 13. Regarding attitudes toward epilepsy, most participants (93.4%) had positive attitudes toward students with epilepsy. There is a strong correlation between experience and attitude toward epilepsy. Most of the participants lacked adequate first aid training and felt unprepared to deal with a child with a seizure.

Conclusion: Most school teachers in Madinah had positive attitudes toward epilepsy patients. Although they had a basic understanding of epilepsy, there were gaps in their knowledge, and they lacked adequate training and confidence in assisting a child with a seizure. Therefore, it is essential to implement education and training programs aimed at improving their knowledge and confidence in dealing with students with epilepsy.

## Introduction

Epilepsy is a prevalent chronic condition and non-communicable neurologic disorder affecting approximately 50 million people worldwide, and nearly 80% of them live in developing regions [1]. A study in Saudi Arabia in 2001 showed prevalence rates of 6.54/1000 population [2]. Epilepsy is one of the diseases that can be associated with poor quality of life as a sequence of seizures, and it is associated with cognitive and psychiatric disorders and medication adverse effects [3].

Healthcare professionals and individuals with epilepsy often face problems [4]. Some of the major problems faced by both include a variety of challenges that can significantly impact their overall health and well-being such as poor knowledge in the community, lack of awareness, cultural beliefs, and stigma [5,6]. Children with epilepsy face erroneous attitudes mainly in social environments like schools [7]. Some students with epilepsy do not perform well in school and others present with difficulties in learning and social interactions [5]. The challenges that a child with epilepsy may face are not limited to the condition itself, but can also be influenced by psychological factors such as the educator's qualifications, negative attitudes and expectations from teachers, parents, and peers, and the child's own low self-esteem [4].

Globally, teachers have misconceptions about epilepsy and its management [8]. Studies in Korea and Brazil found that teachers play an important role in influencing the educational performance of children, particularly those with epilepsy [9,10]. In Saudi Arabia, teachers are considered social leaders and role models, and that gives them a vital role to play in the development of an attitude toward any disease of the school child, As teachers play a crucial role in the education and well-being of these children, health education programs should be developed to improve their attitude toward and knowledge of epilepsy [11]. In addition, there is a lack of a proper plan of action to deal with students with epilepsy which has a negative impact on them and may even endanger their lives. Health education programs on epilepsy should be designed to educate school teachers and thereby, to eventually create a well‐informed and tolerant community [5]. However, despite the significant impact of teacher in the lives of these children, there is limited research on this topic in Saudi Arabia, especially in Madinah city, highlighting the need for further investigation and educational program development to enhance the quality of life for those with epilepsy. For these reasons, this study was conducted to assess the knowledge of, and attitude toward, epilepsy among school teachers in Madinah city, Saudi Arabia. In addition, this study serves as a base for future research and supports the need for the development of educational programs that will have a major impact on the quality of life of patients with epilepsy.

## Materials and methods

A descriptive cross-sectional study using an online self-administered questionnaire was conducted between March 1, 2022, and November 30, 2022, in Madinah, Saudi Arabia. Convenience sampling techniques were used to recruit study participants.

A total of 394 teachers were recruited from Madinah schools. Study participants were asked to complete an online survey. The survey was distributed via social media platforms (Twitter/X (Twitter, Inc., San Francisco, California, United States), WhatsApp (Meta Platforms, Inc., Menlo Park, California, United States), etc.). Since all the participants volunteered to participate in the study, there was no need for written informed consent. The study included primary, middle, and high school teachers from both genders who are actively teaching students in Madinah. The study excluded teachers who were not able to read and communicate in Arabic as the survey was written in Arabic.

The questionnaire was adapted using scales that have previously been verified in other research studies in Turkey and Saudi Arabia [5,12,13]. It is divided into two sections and contains 16 items. The first section involved demographic information, including age, gender, academic qualifications, and years of experience. The second section included general information about epilepsy to assess their knowledge and attitudes. The sample size was calculated using G*Power software following parameters: alpha = 0.05, power = 0.95, and low effect size = 0.2. The minimum total sample size needed for this study was 260. To prevent attrition and non-response rates, the sample size was increased by 20% and the total sample size was calculated to be 312. This was finally increased 394. The data were analyzed using the IBM SPSS Statistics for Windows, Version 26.0 (Released 2019; IBM Corp., Armonk, New York, United States) was used for data analysis. Categorical variables and participants' responses were represented as frequency and percentages.

Data normality was tested using the Kolmogorov-Smirnov test. The association of knowledge score and attitude score toward epilepsy with sociodemographic characteristics of participants was conducted using the Mann-Whitney U test (for two groups); and the Kruskal Wallis H test (for more than two groups). A p-value of less than 0.05 was considered statistically significant, and the confidence interval was 95%.

## Results

The sample consisted of 394 employees/teachers of schools of Madinah. Table 1 illustrates the sociodemographic characteristics of the sample; 36.3% (n=143) reported their gender as male and 64.7% (n = 251) as female, and approximately 56% of the participants were aged 40-49 years. Also, 85.8% of the sample were married and most of the participants (85.8%) reported completing a university bachelor's degree as their highest level of education. Besides, 47% of the sample had more than 21 years of experience. Most of the participants worked in public schools (94.9%).

**Table 1:** Sociodemographic characteristics of the sample (N=394)

Characteristics	Frequency	Percentages
Gender		
Male	143	36.3%
Female	251	64.7%
Age (years)		
Less than 30	9	2.3%
30-39	58	14.7%
40-49	221	56.1%
More than or equal to 50 years	106	26.9%
Nationality		
Saudi	98.5	98.5%
Non-Saudi	1.5	1.5%
Marital status		
Single	26	6.6%
Married	338	85.8%
Divorced/widowed	30	7.6%
Educational Level		
Secondary Stage	6	1.5%
Diploma	19	4.8%
Bachelor's degree	338	85.8%
Master's degree	28	7.1%
Doctorate (PhD)	3	0.8%
Position/Designation		
Teacher	344	87.3%
Student Advisor	17	4.3%
Deputy Principal	17	4.3%
Director	16	4.1%
Teaching-Experience		
Less than or equal to 5 years	17	4.3%
6-10 years	52	13.2%
11-15 years	77	19.5%
16-20 years	62	15.7%%
21-25	94	23.9%
More than 25 years	92	23.4%
School Type		
Public school	374	94.9%
Private school	20	5.1%

Table 2 shows the participants' experience with epilepsy. Most respondents (90.4%) positively answered the item “Would you like to have more information about how to respond when a student is having a seizure?”. Similarly, 86% positively responded to the item “Would you like to have more general knowledge about epilepsy?”, 37.3% positively answered the question “Will you be prepared to handle a seizure if one of your students had a fit during class?”, 32.2% positively responded to the question “Have you ever dealt with a person having epilepsy?”, and 24.9% replied positively to the question “Have you been a teacher of a student with epilepsy?”. Only 11.4% thought that they had sufficient training in first-aid management of seizures, and 9.4% reported there was a member of their family who has epilepsy. Only 7.4% of respondents reported being current teachers of students with epilepsy, and they were aware of the different types of seizures. Surprisingly, only 4.1% of participants had received adequate training about seizure management and epilepsy in their education curricula.

**Table 2:** Responses to questions regarding experience with epilepsy (N=394)

Experience items	Yes		Not sure		No	
	N	%	N	%	N	%
Have you ever dealt with a person with epilepsy?	127	32.2%	34	8.6%	233	59.1%
Does any member of your family have epilepsy?	37	9.4%	7	1.8%	350	88.8%
Have you been a teacher of a student with epilepsy?	98	24.9%	21	5.3%	275	69.8%
Are you currently a teacher of a student with epilepsy?	29	7.4%	16	4.1%	349	88.6%
Are you aware of the life circumstances of persons with epilepsy?	106	26.9%	78	19.8%	210	53.3%
Will you be prepared to handle a seizure if one of your students had a fit during class?	147	37.3%	133	33.8%	114	28.9%
Do you think you have sufficient training in first-aid management of seizures?	45	11.4%	62	15.7%	287	72.8%
Are you aware of the different types of seizures and what they look like?	29	7.4%	58	14.7%	307	77.9%
Would you like to have more general knowledge about epilepsy?	339	86.0%	21	5.3%	34	8.6%
Would you like to have more information about how to respond when a student is having a seizure?	356	90.4%	18	4.6%	20	5.1%
Have you received adequate training about seizure management and epilepsy in your teaching training?	16	4.1%	12	3.0%	366	92.9%

**Figure 1:**
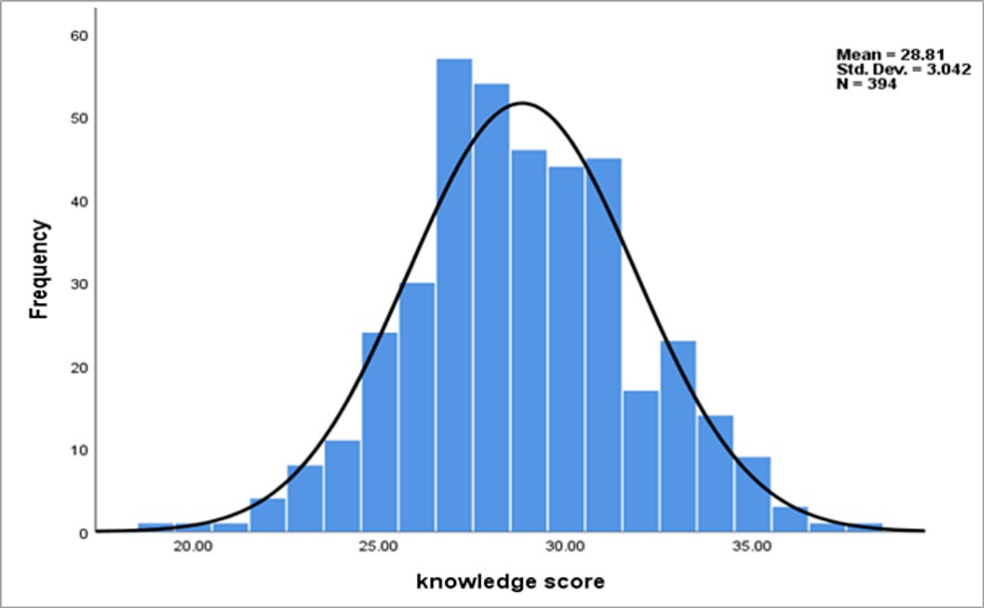
Frequency distribution of knowledge scores about epilepsy

Table 3 presents the 13 items of knowledge score about epilepsy together with the percentage of participants who answered each item on a three-point Likert scale. A high proportion (93.4%) of participants agreed to the correct knowledge item “epilepsy is not a contagious disease", while a high proportion (68%) of participants agreed with the wrong knowledge item “individuals with epilepsy are accident-prone". The mean score for epilepsy knowledge was 28.8 (SD±3.04), which indicated the participants had good knowledge about epilepsy (Figure 1).

**Table 3:** Responses to questions regarding knowledge about epilepsy (N=394)

Knowledge items	Agree		Not sure		Disagree	
	N	%	N	%	N	%
Individuals with epilepsy also face developmental delays.	7	1.8%	9	2.3%	378	95.9%
The individual with epilepsy does not possess a normal life expectancy.	33	8.4%	121	30.7%	240	60.9%
You can expect the condition of a person with epilepsy to deteriorate.	162	41.1%	144	36.5%	88	22,3%
When their seizures are controlled by medication, persons with epilepsy are just like anyone else.	306	77.7%	73	18.5%	15	3.8%
Individuals with epilepsy can cope with a 40-hour work week.	82	20.8%	205	52.0%%	107	27.2%
Persons with epilepsy can safely participate in strenuous activity.	65	16.5%	135	34.3%	194	49.2%
Persons with epilepsy can safely operate machinery.	75	19.0%	142	36.0%	177	44.9%
Individuals with epilepsy are accident-prone.	268	68.0%	95	24.1%	31	7.9%
Epilepsy is not a contagious disease.	368	93.4%	18	4.6%	8	2.0%
The offspring of parents with epilepsy will also have epilepsy	65	16.5%	188	47.7%	141	35.8%
Persons with epilepsy prefer to live with others of similar characteristics.	47	11.9%	166	42.1%	181	45.9%
Children with epilepsy in regular classes have an adverse effect on the other children.	75	19.0%	114	28.9%	205	52.0%
Epilepsy and epilepsy medications can have a significant effect on the affected students’ mood, memory, and learning.	156	39.6%	189	48.0%	49	12.4%

**Figure 2:**
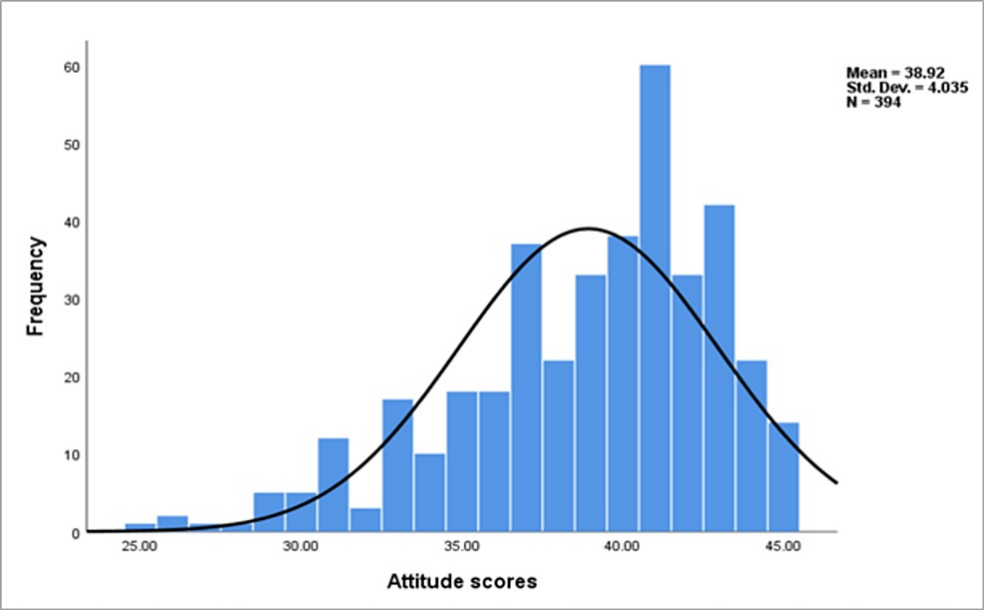
Frequency distribution of attitude scores toward epilepsy

Table 4 presents the 15 items of attitude scores toward epilepsy together with the percentage of participants who answered each item. A high proportion (93.4%) of the participants responded "agree" to the positive attitude item “persons with epilepsy have the same rights as all people", while a median proportion (60.7%) responded "agree" to the negative attitude item “parents should expect of their child who has epilepsy what they expect of other children". The mean attitude score for the sample was 38.9 (SD±4.03), which indicated the participants had a positive attitude toward epilepsy (Figure 2).

**Table 4:** Responses to questions regarding attitude toward epilepsy (N=394)

Attitude items	Agree		Not sure		Disagree	
	N	%	N	%	N	%
Persons with epilepsy have the same rights as all people.	368	93.4	12	3.0	14	3.6
Equal employment opportunities should be available to individuals with epilepsy.	332	84.3	34	8.6	28	7.1
Insurance companies should not deny insurance to individuals with epilepsy.	346	87.8	32	8.1	16	4.1
Persons with epilepsy should be prohibited from driving.	151	38.3	130	33.0	113	28.7
Persons with epilepsy should not be prohibited from marrying.	328	83.2	25	6.3	41	10.4
The individual with epilepsy should not be prevented from having children.	320	81.2	28	7.1	46	11.7
The onset of epileptic seizures in a spouse is a sufficient reason for divorce.	29	7.4	110	27.9	255	64.7
Persons with epilepsy are a danger to the public.	17	4.3	34	8.6	343	87.1
Persons with epilepsy are more likely to develop and express criminal tendencies than other people.	22	5.6	70	17.8	302	76.6
Parents should expect of their child who has epilepsy what they expect of other children.	239	60.7	81	20.6	74	18.8
The responsibility for educating children with epilepsy rests on the community.	240	60.9	77	19.5	77	19.5
Schools should not place children with epilepsy in regular classrooms.	69	17.5	61	15.5	264	67.0
Children need to be protected from classmates who have epilepsy.	99	25.1	75	19.0	220	55.8
Children with epilepsy should attend regular public schools.	307	77.9	56	14.2	31	7.9
Families of children with epilepsy should not be provided with supportive social services.	45	11.4	51	12.9	298	75.6

Table 5 shows an association between knowledge scores about epilepsy and with sociodemographic characteristics of participants. The results showed there was a significant difference between the rank of mean score of participants who worked in public schools and those who worked in private schools (p = 0.02). The participants in private schools showed higher knowledge scores about epilepsy (253.13) compared to participants from public schools (192.42).

**Table 5:** Association between knowledge about epilepsy and sociodemographic characteristics of participants ^a ^Mann-Whitney U test, ^b^ Kruskal Wallis test, * P-value <0.05 is statistically significant.^ ^

Characteristics	Mean Rank	P-value
Gender ^a^		
Male	198.35	0.758
Female	194.69	
Age ^b^		
Less than 30 years	227.83	0.869
30-39 years	195.23	
40-49 years	195.54	
More than or equal to 50 years	196.53	
Nationality ^a^		
Saudi	196.03	0.963
Non-Saudi	193.92	
Marital status ^b^		
Single	196.32	0.885
Married	196.29	
Divorced/widowed	185.62	
Educational Level ^b^		
Secondary Stage	147.63	0.910
Diploma	198.14	
Bachelor's degree	193.93	
Master's degree	204.85	
Doctorate (PhD)	206.33	
Position/designation ^b^		
Teacher	195.54	0.723
Student Advisor	208.79	
Deputy Principal	168.75	
Director	207.25	
Teaching Experience ^b^		
Less than or equal to 5 years	221.00	0.369
6-10 years	208.49	
11-15 years	195.42	
16-20 years	200.07	
21-25	186.97	
More than 25 years	173.02	
School Type ^a^		
Public school	192.92	0.020*
Private school	253.13	

Table 6 illustrates the association between knowledge score about epilepsy with participants' experience of epilepsy. The results showed there was a significant difference between the rank of mean to knowledge about epilepsy by the different responses of participants on the item "Are you aware of the life circumstances of persons with epilepsy?" (p = 0.016). Post hoc comparisons found there was a significant difference in rank of the mean to knowledge about epilepsy favoring those who answered "yes", "not sure" (p = .007) compared to those who answered "no". Also, there was a significant difference between the rank of mean knowledge about epilepsy on the item "are you aware of the different types of seizures and what they look like? (p = 0.001). Post hoc comparisons found there was a significant difference in difference in rank of the mean to knowledge about epilepsy favoring those who answered "yes", not sure" (p = .001, p = .018, respectively) compared to those who answered "no".

**Table 6:** Association between knowledge about epilepsy with participants' experience of epilepsy

Experience items	Category	Mean Rank	P-value
Have you ever dealt with a person with epilepsy?	No	187.24	0.465
	Not sure	204.87	
	Yes	200.19	
Does any member of your family have epilepsy?	No	196.00	0.724
	Not sure	197.57	
	Yes	211.72	
Have you been a teacher of a student with epilepsy?	No	198.47	0.780
	Not sure	184.88	
	Yes	191.45	
Are you currently a teacher of a student with epilepsy?	No	195.58	0.105
	Not sure	126.59	
	Yes	180.95	
Are you aware of the life circumstances of persons with epilepsy?	No	182.41	0.016*
	Not sure	208.94	
	Yes	218.97	
Will you be prepared to handle a seizure if one of your students had a fit during class?	No	183.86	0.261
	Not sure	194.63	
	Yes	206.79	
Do you think you have sufficient training in first-aid management of seizures?	No	189.44	0.123
	Not sure	213.52	
	Yes	218.27	
Are you aware of the different types of seizures and what they look like?	No	179.13	0.001*
	Not sure	216.30	
	Yes	248.14	
Would you like to have more general knowledge about epilepsy?	No	173.50	0.431
	Not sure	213.37	
	Yes	194.86	
Would you like to have more information about how to respond when a student is having a seizure?	No	166.68	0.513
	Not sure	193.75	
	Yes	197.14	
Have you received adequate training about seizure management and epilepsy in your teaching training?	No	194.18	0.367
	Not sure	239.75	
	Yes	204.44	

Table 7 illustrates the association between attitude score toward epilepsy and the sociodemographic characteristics of participants. The results showed there was a significant difference between the mean scores for attitude toward epilepsy by the different ages of participants (p = 0.042). Post hoc comparisons found there was a significant difference in the rank of the mean to attitude toward epilepsy scores favoring those in the age group of 40-49 years, and ≥ 50 years (p = 0.037, p = 0.01, respectively) compared to those in the age group of 30-39 years. Also, there was a significant difference between the mean scores on attitude toward epilepsy among the different social statuses of participants (p = 0.014). Post hoc comparisons found there was a significant difference in the mean rank of attitude toward epilepsy scores between single and married participants (p = 0.006), favoring married participants. Finally, there was a significant difference between the mean scores of attitude toward epilepsy and different educational levels (p < 0.001). Post hoc comparisons found there was a significant difference in rank of the mean to attitude toward epilepsy scores favoring those who had a bachelor's degree (p = 0.003), and master's degree (p < 0.001) compared to those who had a secondary level education, and there was a significant difference in rank of the mean to attitude toward epilepsy scores favoring those who had a master's degree compared to those who had a diploma (p < 0.001), and bachelor's degree (p < 0.001).

**Table 7:** Association between attitude toward epilepsy with sociodemographic characteristics of participants. ^a^ Mann-Whitney U test, ^b^ Kruskal Wallis test, * P-value <0.05 is statistically significant.

Characteristics	Mean Rank	P-value
Gender ^a^		
Male	192.33	0.874
Female	194.19	
Age ^b^		
Less than 30 years	150.22	0.042*
30-39 years	159.82	
40-49 years	202.33	
More than or equal to 50 years	197.82	
Nationality ^a^		
Saudi	192.56	0.930
Non-Saudi	188.58	
Marital status ^b^		
Single	136.96	0.014*
Married	199.05	
Divorced/widowed	173.55	
Educational Level ^b^		
Secondary stage	57.50	0.001
Diploma	146.74	
Bachelor's degree	191.68	
Master's degree	268.38	
Doctorate (PhD)	210.00	
Position ^b^		
Teacher	189.07	0.697
Student Advisor	217.24	
Deputy Principal	212.83	
Director	192.18	
Teaching Experience ^b^		
Less than or equal to 5 years	185.09	0.754
6-10 years	180.78	
11-15 years	202.43	
16-20 years	206.56	
21-25	184.89	
More than 25 years	195.12	
School Type ^a^		
Public school	193.13	0.636
Private school	181.10	

Table 8 illustrates the association between attitude score toward epilepsy with participants’ experience of epilepsy. The results showed there was a significant difference between the rank of mean scores to attitude toward epilepsy of participants by the response to the item “Are you aware of the life circumstances of persons with epilepsy?” (p = 0.004). Post hoc comparisons found there was a significant difference in the mean rank to attitude toward epilepsy scores favoring those who answered “yes” (p = 0.002), and “not sure”(p = 0.032) compared to those who answered “no”. Also, there was a significant difference in the rank of mean scores to attitude toward epilepsy on the item “Will you be prepared to handle a seizure if one of your students had a fit during class?” (p = 0.01). Post hoc comparisons found there was a significant difference favoring those who answered, “not sure” (p = 0.003) compared those who answered “no”. Moreover, there was a considerable difference between the rank of mean scores to attitude toward epilepsy on the item “Would you like to have more general knowledge about epilepsy?” p < 0.001. Post hoc comparisons found there was a significant difference between those who answered “no” and those who answered “yes” (p < 0.001) favoring participants who answered “yes”. Finally, there was a significant difference between the rank of mean scores to attitude toward epilepsy on the item “Would you like to have more information about how to respond when a student is having a seizure?” p = 0.001. Post hoc comparisons found there was a significant difference between those who answered “no” and those who answered “yes” (p < 0.001) favoring participants who answered “yes”.

**Table 8:** Association between attitude toward epilepsy with participants' experience of epilepsy

Experience items	Category	Mean Rank	P-value
Have you ever dealt with a person with epilepsy?	No	187.88	0.423
	Not sure	202.63	
	Yes	203.07	
Does any member of your family have epilepsy?	No	191.27	0.790
	Not sure	189.64	
	Yes	204.34	
Have you been a teacher of a student with epilepsy?	No	194.64	0.791
	Not sure	179.36	
	Yes	189.49	
Are you currently a teacher of a student with epilepsy?	No	195.63	0.177
	Not sure	145.44	
	Yes	181.41	
Are you aware of the life circumstances of persons with epilepsy?	No	175.83	0.004*
	Not sure	221.81	
	Yes	204.26	
Will you be prepared to handle a seizure if one of your students had a fit during class?	No	171.00	0.010*
	Not sure	214.50	
	Yes	190.74	
Do you think you have sufficient training in first-aid management of seizures?	No	191.34	0.940
	Not sure	196.55	
	Yes	194.23	
Are you aware of the different types of seizures and what they look like?	No	192.99	0.697
	Not sure	198.03	
	Yes	176.91	
Would you like to have more general knowledge about epilepsy?	No	117.38	0.001
	Not sure	168.17	
	Yes	204.59	
Would you like to have more information about how to respond when a student is having a seizure?	No	106.20	0.001*
	Not sure	163.70	
	Yes	200.28	
Have you received adequate training about seizure management and epilepsy in your teaching training?	No	193.47	0.301
	Not sure	217.80	
	Yes	154.91	

## Discussion

Epilepsy is a common chronic neurological disease in children. As children spend a considerable amount of time at school, teachers’ knowledge of and attitude toward epilepsy, and experience in seizures and first aid are crucial and play a major role in the well-being, behavior, and development of children with epilepsy. Previous studies were conducted nationally and internationally to evaluate teachers’ knowledge of and attitudes toward epilepsy over the years. We conducted this study to evaluate current teachers’ knowledge and attitudes about epilepsy in Madinah.

The current study revealed that around a third of our teachers have dealt with epileptic patients and a quarter of teachers have a current student with epilepsy. Similarly, about 37% of teachers in our study think they can handle a seizure if happens in the class which is consistent with the result of previous studies in Kuwait, Khamis Mushate, and Riyadh [14,5,4]. Moreover, only 7.4% reported being aware of the different types of seizures, which is similar to a previous study in Kuwait [14]. The majority of our teachers didn’t receive adequate information about epilepsy and its management during their training curriculum, which is in line with the result of previous studies in Mecca [15], Kuwait [14], and Sudan [16], and only 11.4% of our sample had the sufficient seizure’s first aid similar to school teachers in Jeddah [17]. However, most of our teachers are willing to know more information about epilepsy and how to respond when a student is having a seizure. These results are in keeping with previous responses from teachers in Kuwait, Yanbu, and Khamis Mushate [5,14,18]. This demonstrates the continuous lack of first aid training among teachers and the ongoing need to initiate epilepsy awareness and training programs for schoolteachers to improve their knowledge and confidence in dealing with epilepsy patients, which likely will have a major impact on the student’s life and development.

In general, our study participants had a good knowledge of epilepsy, which is consistent with the result of a recent similar study in Jeddah and with the general improvement of knowledge seen among Saudi teachers over the recent years [17]. The majority of our participants (96%) disagreed with the statement that individuals with epilepsy are mentally retarded, which is similar to the results of previous studies conducted in Yanbu, Kuwait, and Kuwait Mushait (96%, 84%, and 83%, respectively) [18,14,5]. This result does not accord with previous studies in Niger [19], Riyadh [4], and Egypt [20] that surprisingly showed 57%, 47%, and 58% of teachers, respectively, believe that epilepsy is a disease observed always in a mentally impaired person. Moreover, similar to a study conducted in Yanbu [18], our study showed 93% of respondents correctly answered the knowledge item “epilepsy is not a contagious disease,” which is slightly better than the Kuwait study where 82.4% of the respondents correctly answered the same question. Previous studies in Egypt [20] and Brazil [21] showed better results with only 1.6% and 1%, respectively, thinking that epilepsy is a contagious disease. Of our respondents, 78% correctly answered the statement that a child with epilepsy is like anyone else just after seizures are controlled, which is better than 65.8% among Kuwait teachers. However, poor epilepsy knowledge was observed with only 36% of our teachers disagreeing with the statement that “the offspring of parents with epilepsy will also have epilepsy”, which is consistent with previous Kuwait [14] and Jordan [22] studies results. Similarly, only 40% of our participants think correctly that epilepsy medications have a significant effect on students' mood, memory, and learning, compared to 70% in Yanbu and 51%) in Kuwait [5,14,18].

Our study showed no significant association between the knowledge score and the teachers’ characteristics including age, sex, and years of experience. A similar result was seen in previous studies conducted in Mecca [15] and Yanbu [18]. However, private school teachers showed a significantly higher median knowledge score when compared with public school teachers, which is different than previous studies conducted in Arar City in Saudi Arabia and Egypt [23,20]. Moreover, there was an direct association of the knowledge score about epilepsy with self-reported experience and answered yes to items "Are you aware of life circumstances of persons of epilepsy" and "Are you aware of different types of seizures". This is consistent with results from previous studies where teachers' previous experience with epilepsy was found to be associated with a better knowledge score [8].

A significant majority (67%) of the participants displayed a positive outlook and disagreed with the notion that children with epilepsy should not be integrated into regular classrooms. Our findings align with a study conducted by Alqahtani, in which the majority of school teachers (80%) disagreed with the idea of segregating children with epilepsy [5]. Similar results were observed in previous studies conducted in Egypt (72.5%) and Riyadh (85.7%) [20,4]. Likewise, 93% of teachers agreed that individuals with epilepsy have equal rights, which corresponds to the results of a study conducted in Kuwait [14]. Furthermore, 83% of our participants agreed that individuals with epilepsy should not be prohibited from getting married, which is significantly higher than the percentages reported by teachers in Yanbu (41%) and Taif (78%) [18,8]. However, 38% of our respondents believed that individuals with epilepsy should be prohibited from driving, contrasting with the findings from Kuwait (14%), where only 15.7% of teachers held the same view [14].

Regarding attitudes toward epilepsy, participants over the age of 40 exhibited significantly higher mean attitude scores compared to those under 40 years, while married teachers displayed more positive attitudes compared to their single counterparts. This finding is consistent with a previous study conducted in Kuwait, which also found a significantly more positive attitude among married teachers [14]. Additionally, teachers with higher levels of education displayed higher attitude scores, which aligns with the study conducted in Kuwait, indicating a more positive attitude toward epilepsy among teachers with higher education. However, a study conducted in Niger did not find a significant association between higher education and a positive attitude toward epilepsy [19]. Participants who reported prior experience and awareness of the challenges faced by individuals with epilepsy demonstrated significantly higher attitude scores. This result is similar to previous studies [14], which also identified significant differences in attitudes between teachers with and without previous experience with epilepsy. Similarly, individuals who expressed a desire for more general knowledge about epilepsy and information on how to respond when a student is having a seizure exhibited higher attitude scores. This finding is consistent with the study conducted in Kuwait, which revealed a strong interest among participant teachers in acquiring more education about epilepsy [14].

## Conclusions

The prevalence of epilepsy in Saudi Arabia highlights the need for healthcare providers to address the challenges faced by individuals with this condition. Even though the teachers showed better knowledge about the disease and expressed a more positive attitude than in earlier years, they still lacked first aid skills and found it challenging to assist a student who was experiencing a seizure. Students with epilepsy are negatively impacted by this lack of planning, which could possibly put their lives in jeopardy. Our teachers are enthusiastic to learn more about epilepsy and willing to undergo first aid training. Therefore, school teacher education programs on epilepsy should be developed and incorporated into teachers’ training curricula to ensure a safe environment for individuals with epilepsy. 
